# A Comparison of SGLT2 or DPP-4 Inhibitor Monotherapy vs Placebo for Type 2 Diabetes in Adolescents vs Young Adults

**DOI:** 10.1210/jendso/bvaf085

**Published:** 2025-06-24

**Authors:** Lori M Laffel, Thomas Danne, Georgeanna J Klingensmith, Elke Schueler, Igor Tartakovsky, Laurieann Nessralla, Philip Zeitler, Steven Willi

**Affiliations:** Harvard Medical School, Joslin Diabetes Center, Boston, MA 02215, USA; Diabetes Centre for Children and Adolescents, Auf der Bult Kinder- und Jugendkrankenhaus, Hannover 30173, Germany; Barbara Davis Center, University of Colorado, Aurora, CO 80045, USA; mainanalytics GmbH, Sulzbach 65843, Germany; Clinical Development & Medical Affairs, Boehringer Ingelheim International GmbH, Ingelheim 55218, Germany; Global Patient Safety and Pharmacovigilence, Boehringer Ingelheim Pharmaceuticals, Inc., Ridgefield, CT 06877, USA; Children’s Hospital Colorado, University of Colorado Anschutz Medical Campus, Aurora, CO 80045, USA; Diabetes Center for Children, Children’s Hospital of Philadelphia, Philadelphia, PA 19104, USA

**Keywords:** adolescents/children, DPP inhibitor, empagliflozin, monotherapy, SGLT2 inhibitor, type 2 diabetes

## Abstract

**Context:**

There is an unmet need for type 2 diabetes (T2D) treatments in addition to metformin and insulin for adolescents. This is due to the challenges of monotherapy in youth with T2D and need for treatment escalation to maintain glycemic control in youth generally more so than in young adults.

**Objective:**

We assessed the efficacy and safety of sodium-glucose co-transporter-2 (SGLT2) or dipeptidyl peptidase-4 (DPP-4) inhibitor monotherapies in adolescents and young adults with T2D not on active therapy.

**Methods:**

Drug-naïve adolescents and those not on active therapy received the SGLT2 inhibitor empagliflozin, the DPP-4 inhibitor linagliptin, or placebo for 26 weeks; young adults with no antidiabetic background therapy received empagliflozin, the DPP-4 inhibitor sitagliptin, or placebo for 24 weeks. The primary endpoint was treatment failure occurrence. Secondary outcomes assessed glycated hemoglobin A1c (HbA1c), fasting plasma glucose, and weight.

**Results:**

Treatment failure rates were similar for empagliflozin and linagliptin vs placebo in adolescents, but significantly reduced with empagliflozin in young adults (*P* = .017). Empagliflozin modestly reduced mean HbA1c vs placebo in adolescents (−0.35% vs 0.41%) compared with greater reductions in young adults (−1.01% vs −0.30%). No new safety signals were identified.

**Conclusion:**

Empagliflozin reduced HbA1c in adolescents and young adults; however, these results highlight the challenges of monotherapy for youth with T2D and need for further studies.

There is an unmet need for type 2 diabetes (T2D) treatments for adolescents in addition to metformin and insulin [1, 2]. The American Diabetes Association Standards of Care recommends the initiation of monotherapy in adolescents at diagnosis of T2D (in addition to lifestyle changes focusing on diet and exercise), with metformin the initial therapy of choice in those with normal renal function [3]. However, monotherapy is not always sufficient to maintain glycemic control. For instance, in the TODAY study, youth with T2D treated with metformin monotherapy had a treatment failure rate of 51.7% with a median time to failure of 10.3 months [4]. Additionally, a retrospective cohort study of 829 adolescents with T2D found that only 25% of youth initially treated with metformin monotherapy underwent treatment escalation within 5 years, a number which may be impeded by a lack of oral treatment options [5].

DINAMO was a phase 3 trial assessing the efficacy and safety of the sodium-glucose co-transporter-2 (SGLT2) inhibitor empagliflozin and the dipeptidyl peptidase-4 (DPP-4) inhibitor linagliptin, each vs placebo, in participants with T2D aged 10-17 years, previously treated with metformin and/or insulin, and showed that empagliflozin provided durable, clinically relevant improvements in glycemic control [6]. DINAMO MONO, an ancillary study to DINAMO, investigated the same treatments [6] in participants with T2D who were drug-naïve or not on active therapy. The recruitment into DINAMO MONO was stopped prematurely based on regulator feedback communicating a change of view on the value of a monotherapy pediatric trial, and difficulties recruiting treatment-naïve participants leading to a lower final sample size than targeted.

This study examined the efficacy and safety of empagliflozin or linagliptin vs placebo in adolescents from DINAMO MONO pooled with drug-naïve adolescents from DINAMO, compared with young adults aged 18-35 years with no antidiabetic background therapy treated with empagliflozin or the DDP-4 inhibitor sitagliptin vs placebo. In this post hoc analysis, we aimed to highlight the challenges of managing adolescents, compared with young adults, with T2D on monotherapy.

## Materials and Methods

### Trial Design

DINAMO MONO was operationally under the same protocol as DINAMO. The clinical trial protocol and statistical analysis plan for DINAMO and DINAMO MONO, and results from the DINAMO trial have been published previously [6].

The key inclusion criteria for DINAMO MONO were adolescents aged 10-17 years at randomization with a documented diagnosis of T2D, and insufficient glycemic control (HbA1c ≥6.5% and ≤9.0%) at baseline; participants were drug-naïve or not on active treatment (including, at the investigator's discretion, discontinuation of metformin due to intolerance [or previous discontinuation for other reasons] and/or discontinuation of insulin [insulin use had to be ≤8 weeks in total]) prior to or at screening. Inclusion criteria for DINAMO MONO differed from DINAMO in the following ways: firstly, DINAMO MONO required confirmation of T2D at Visit 1A whereas DINAMO required documented diagnosis of T2D for at least 8 weeks at Visit 1A. Secondly, DINAMO MONO required HbA1c ≥6.5% and ≤9.0% at Visit 1A whereas DINAMO required HbA1c ≥6.5% and ≤10.5%. Any participants recruited to DINAMO who were not on active treatment and had a screening HbA1c ≤9.0% therefore met all DINAMO MONO inclusion criteria and outcomes for these participants were pooled with DINAMO MONO.

The young adults comparator participants were participants in a phase 3 trial, EMPA-REG MONO, investigating empagliflozin or sitagliptin vs placebo in adults with T2D [7]. This current analysis included only participants from EMPA-REG MONO who were aged 18-35 years with HbA1c ≥6.5% and ≤9.0% who had not been treated with any antidiabetic drugs within 12 weeks of randomization. Prespecified endpoints from DINAMO MONO were recalculated for the young adult population to allow comparison for this similar population.

Participants from DINAMO + DINAMO MONO (hereinafter referred to as adolescents) were randomized to placebo (n = 9), empagliflozin 10 mg (n = 6), or linagliptin 5 mg (n = 7) once daily. Seventeen participants (placebo: n = 5; empagliflozin: n = 6; linagliptin: n = 6) were recruited for DINAMO MONO and 5 participants (placebo: n = 4; empagliflozin: n = 0; linagliptin: n = 1) were treatment-naïve participants from DINAMO who fulfilled all other DINAMO MONO inclusion criteria. Those receiving empagliflozin 10 mg who did not attain HbA1c <7.0% by Week 12 underwent blinded rerandomization to 10 or 25 mg at Week 14. Young adults were randomized to empagliflozin 10 or 25 mg, sitagliptin 100 mg, or placebo once daily.

### Efficacy

The primary endpoint was treatment failure occurrence up to or at Week 26 or Week 24 for adolescents and young adults, respectively. Treatment failure was defined as use of metformin and/or insulin rescue medication at any time, increase in HbA1c from baseline by 0.5%, or increase in HbA1c to >7.0% in participants with baseline HbA1c <7.0%.

Secondary endpoints included changes in HbA1c, fasting plasma glucose (FPG), and body weight from baseline to Week 26 for adolescents, and to Week 24 for young adults.

For both the adolescents and young adults, all participants treated with empagliflozin were analyzed as 1 treatment group.

### Safety

Safety was assessed until Week 26 in adolescents and until Week 24 in young adults. Adverse events (AEs) of special interest and specific AEs included pancreatitis, hypoglycemia, diabetic ketoacidosis, and events leading to lower limb amputation.

### Statistical Analysis

For the primary endpoint, risk differences of active treatments vs placebo were assessed by an exact 2-sided 90% CI according to Chan and Zhang [8], *P* values were based on Fisher's exact test. Secondary endpoints were summarized using descriptive statistics. All endpoints were analyzed using the modified intention to treat set (all randomized participants treated with at least 1 dose of study medication).

## Results

### Baseline Characteristics

The adolescents were mostly females from North America around 14 years of age, whereas the young adults were mostly males from Asia around 30 years of age. In adolescents baseline values for HbA1c were 7.1% for placebo, 6.8% for empagliflozin, and 7.3% for linagliptin treatment arms. In young adults baseline values for HbA1c were 7.5% for placebo, 7.6% for empagliflozin, and 8.0% for sitagliptin treatment arms. Other baseline characteristics were generally balanced across the treatment groups (Table 1).

**Table 1.** Baseline characteristics of adolescents vs young adults

	Adolescents*^a^* (N = 22)			Young adults (N = 30)		
	Placebo (n = 9)	Empagliflozin (n = 6)	Linagliptin (n = 7)	Placebo (n = 8)	Empagliflozin (n = 17)	Sitagliptin (n = 5)
Age, years, mean (SD)	14.2 (2.0)	14.8 (1.5)	13.9 (3.1)	32.0 (2.5)	30.5 (4.1)	28.6 (3.7)
Sex, n (%)						
Male	3 (33.3)	0 (0.0)	1 (14.3)	6 (75.0)	14 (82.4)	4 (80.0)
Female	6 (66.7)	6 (100.0)	6 (85.7)	2 (25.0)	3 (17.6)	1 (20.0)
Region, n (%)						
North America	7 (77.8)	6 (100.0)	6 (85.7)	5 (62.5)	4 (23.5)	1 (20.0)
South America	0 (0.0)	0 (0.0)	0 (0.0)	0 (0.0)	0 (0.0)	0 (0.0)
Europe	2 (22.2)	0 (0.0)	0 (0.0)	0 (0.0)	1 (5.9)	0 (0.0)
Asia	0 (0.0)	0 (0.0)	1 (14.3)	3 (37.5)	12 (70.6)	4 (80.0)
Race, n (%)						
White	6 (66.7)	1 (16.7)	1 (14.3)	1 (12.5)	4 (23.5)	1 (20.0)
Black or African American	3 (33.3)	5 (83.3)	4 (57.1)	2 (25.0)	0 (0.0)	0 (0.0)
Asian	0 (0.0)	0 (0.0)	1 (14.3)	5 (62.5)	13 (76.5)	4 (80.0)
Other	0 (0.0)	0 (0.0)	1 (14.3)	0 (0.0)	0 (0.0)	0 (0.0)
Time since diabetes diagnosis						
<1 year	6 (66.7)	4 (66.7)	6 (85.7)	2 (25.0)	12 (70.6)	3 (60.0)
1-3 years*^b^*/1-5 years*^c^*	3 (33.3)	2 (33.3)	1 (14.3)	6 (75.0)	2 (11.8)	2 (40.0)
>3 years*^b^*/>5 years*^c^*	0 (0.0)	0 (0.0)	0 (0.0)	0 (0.0)	3 (17.6)	0 (0.0)
BMI, kg/m^2^, mean (SD)	41.12 (11.68)	35.84 (9.46)	34.91 (5.74)	31.28 (5.48)	28.30 (6.62)	27.06 (4.08)
Body weight, kg, mean (SD)	102.61 (27.33)	97.85 (27.06)	97.87 (22.63)	96.69 (26.90)	80.51 (23.75)	78.18 (15.16)
Blood pressure, mmHg, n (%)						
SBP ≥140 or DBP ≥90	1 (11.1)	0 (0.0)	0 (0.0)	3 (37.5)	0 (0.0)	0 (0.0)
SBP <140 and DBP <90	8 (88.9)	6 (100.0)	7 (100.0)	5 (62.5)	17 (100.0)	5 (100.0)
eGFR*^d^*, mL/min per 1.73 m^2^, mean (SD)	116.98 (33.03)	128.19 (25.66)	115.70 (20.03)	—	—	—
HbA1c, %, mean (SD)	7.12 (0.80)	6.83 (0.57)	7.34 (0.69)	7.53 (0.57)	7.60 (0.62)	8.02 (0.86)
FPG, mg/dL, mean (SD)	156.59 (105.31)	106.86 (11.21)	139.80 (23.49)	136.75 (17.84)	148.29 (26.21)	148.20 (41.24)
Fasting C-peptide, nmol/L, mean (SD)	1.11 (0.26)	1.13 (0.54)	1.24 (0.47)	—	—	—

Abbreviations: DBP, diastolic blood pressure; eGFR, estimated glomerular filtration rate; FPG, fasting plasma glucose; HbA1c, glycated hemoglobin; SBP, systolic blood pressure.

^a^Pooled participants from the DINAMO and DINAMO MONO trials.

^b^Time since diabetes diagnosis categories for the DINAMO and DINAMO MONO trials.

^c^Time since diabetes diagnosis categories for the young adults trial.

^d^Based on the Zappitelli combined creatinine and cystatin C prediction equation [9].

### Treatment Failure Occurrence

**Figure 1.**
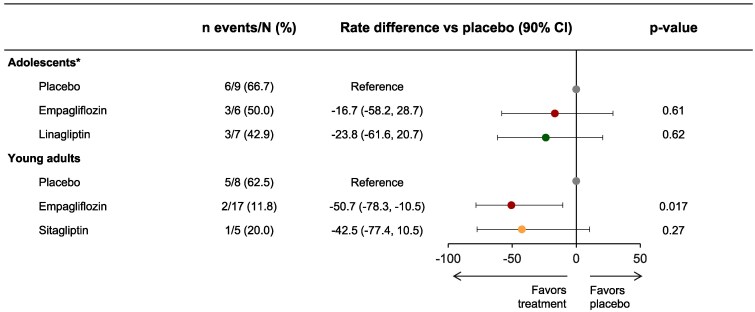
Treatment failure occurrence in adolescents and young adults up to Weeks 26 and 24, respectively. ^∗^Pooled participants from the DINAMO and DINAMO MONO trials.

In adolescents, there was no significant difference in treatment failure occurrence up to Week 26 between empagliflozin and placebo or linagliptin and placebo (Fig. 1). In young adults, empagliflozin significantly reduced treatment failure occurrence up to Week 24 vs placebo (*P* = .017; Fig. 1). There was no significant difference in treatment failure occurrence between sitagliptin and placebo.

### HbA1c Change From Baseline

**Figure 2.**
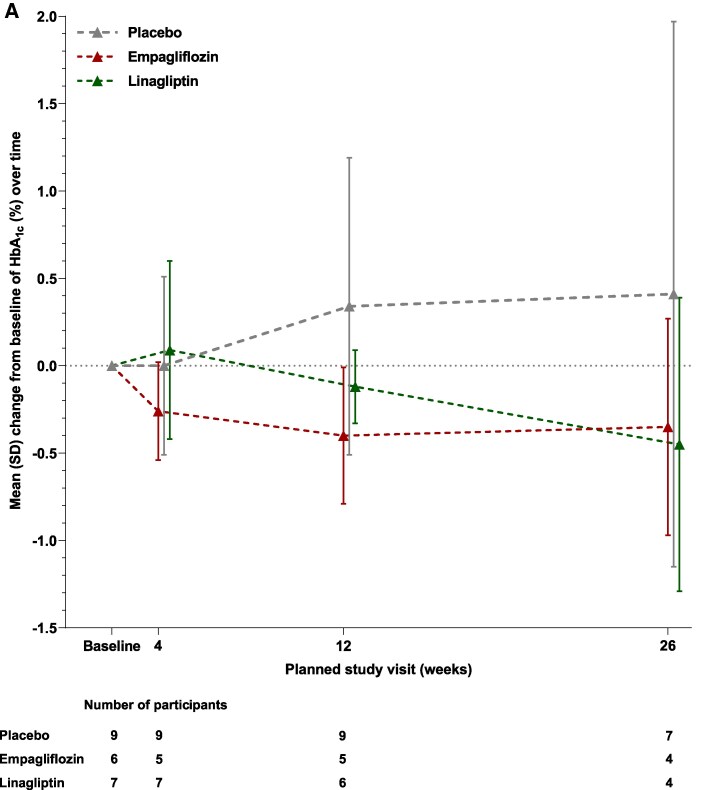

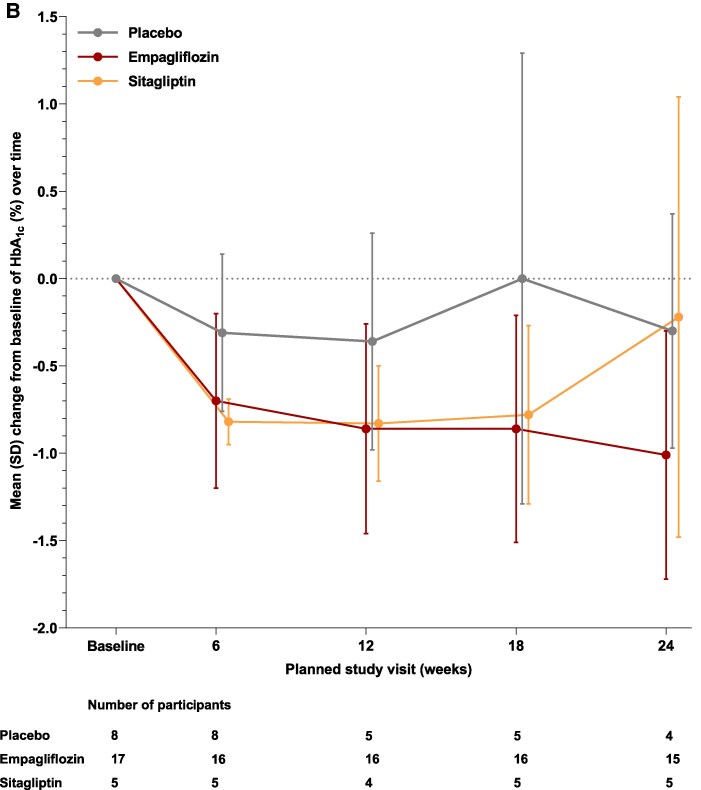

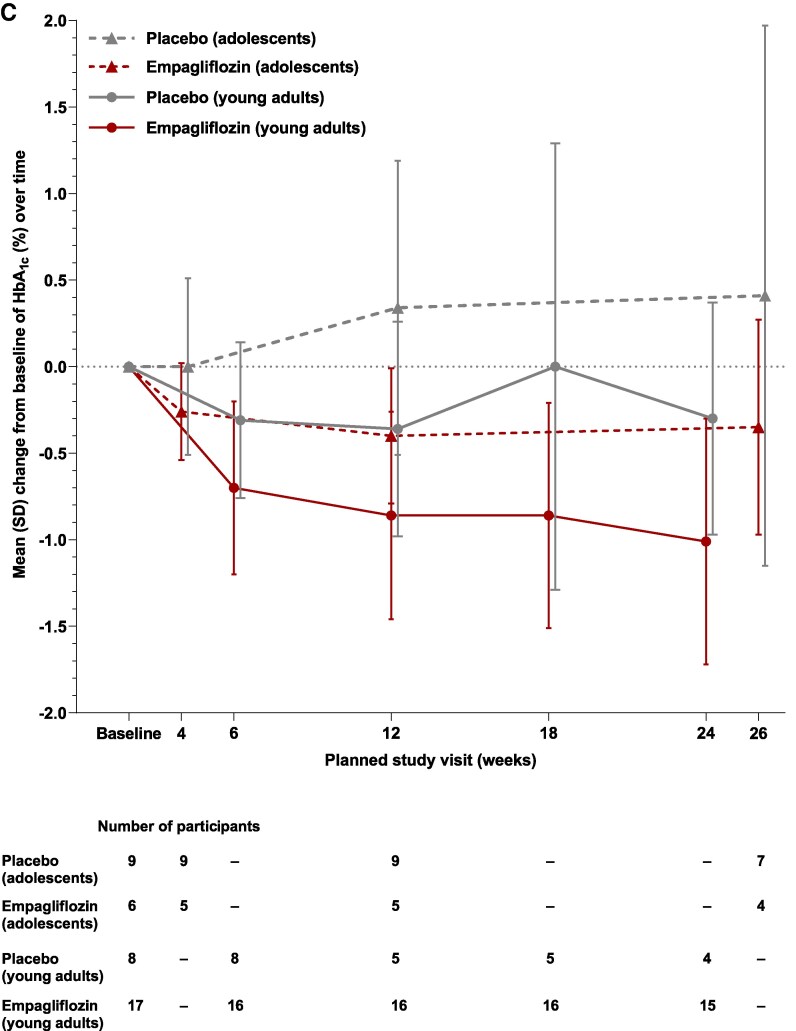
Mean change in HbA1c from baseline (A) to Week 26 in adolescents* for empagliflozin or linagliptin vs placebo and (B) to week 24 in young adults for empagliflozin or sitagliptin vs placebo, and (C) to weeks 26 and 24 in adolescents and young adults, respectively, for empagliflozin vs placebo. Data shown are OC-IR. ^*^Pooled participants from the DINAMO and DINAMO MONO trials.

Abbreviation: HbA1c, glycated hemoglobin.

In adolescents, the mean (SD) change in HbA1c from baseline at Week 26 was 0.41% (1.56) for placebo, −0.35% (0.62) for empagliflozin, and −0.45% (0.84) for linagliptin. In the empagliflozin group, HbA1c decreased by Week 4 and remained below baseline to Week 26. In the linagliptin group, HbA1c increased slightly by Week 4 but then gradually decreased to Week 26 (Fig. 2A). In young adults, mean (SD) change in HbA1c from baseline at Week 24 was −0.30% (0.67) for placebo, −1.01% (0.71) for empagliflozin, and −0.22% (1.26) for sitagliptin. In both the empagliflozin and sitagliptin groups, HbA1c decreased by Week 6 and remained below baseline to Week 24 (Fig. 2B). With empagliflozin treatment, the mean change in HbA1c from baseline to Week 24 was greater in young adults than the change from baseline to Week 26 in adolescents (Fig. 2C).

### FPG Change From Baseline

In adolescents, neither empagliflozin nor linagliptin meaningfully reduced FPG from baseline vs placebo by Week 26 (mean [SD]: placebo, 0.23 mg/dL [26.64]; empagliflozin, 2.64 mg/dL [10.94]; linagliptin, −9.19 mg/dL [34.36]). In young adults, empagliflozin reduced FPG from baseline vs placebo by Week 24, but sitagliptin did not (mean [SD]: placebo, 13.00 mg/dL [11.92]; empagliflozin, −21.60 mg/dL [26.79]; sitagliptin, 9.2 mg/dL [70.74]).

### Body Weight Change From Baseline

In adolescents, neither empagliflozin nor linagliptin meaningfully changed body weight from baseline to Week 26 vs placebo (mean [SD]: placebo, 1.0 kg [8.26]; empagliflozin, 1.45 kg [1.25]; linagliptin, 1.78 kg [2.83]). In young adults, empagliflozin reduced body weight from baseline to Week 24 vs placebo, but sitagliptin did not (mean [SD]: placebo, −1.34 kg [1.65]; empagliflozin, −2.71 kg [4.58]; sitagliptin, 2.66 kg [1.46]).

### Safety

In adolescents, AEs were reported in 66.7%, 100%, and 57.1% of the placebo, empagliflozin, and linagliptin groups, respectively (Table 2). Severe AEs (including pulmonary embolism that caused sudden cardiac arrest, and an upper and lower limb amputation) were reported in 1 participant treated with empagliflozin (see supplemental material [10]). In young adults, AEs were reported in 37.5%, 58.8%, and 100% of the placebo, empagliflozin, and sitagliptin groups, respectively, with no severe AEs reported (Table 2). This analysis found no events of hypoglycemia in adolescents in either the empagliflozin or linagliptin groups up to Week 26, or in young adults in the empagliflozin or sitagliptin groups. There were also no episodes of diabetic ketoacidosis or pancreatitis in either adolescents or young adults.

**Table 2.** Summary of AEs in adolescents and young adults up to week 26 and 24, respectively

AEs, n (%)	Adolescents*^a^* (N = 22)			Young adults (N = 30)		
	Placebo (n = 9)	Empagliflozin (n = 6)	Linagliptin (n = 7)	Placebo (n = 8)	Empagliflozin (n = 17)	Sitagliptin (n = 5)
Any AE	6 (66.7)	6 (100.0)	4 (57.1)	3 (37.5)	10 (58.8)	5 (100.0)
Severe AEs	0 (0.0)	1 (16.7)	0 (0.0)	0 (0.0)	0 (0.0)	0 (0.0)
Drug-related AEs	2 (22.2)	1 (16.7)	0 (0.0)	0 (0.0)	5 (29.4)	1 (20.0)
AE leading to discontinuation	1 (11.1)	2 (33.3)	0 (0.0)	0 (0.0)	0 (0.0)	0 (0.0)
AESIs and specific AEs	3 (33.3)	3 (50.0)	1 (14.3)	0 (0.0)	2 (11.8)	2 (40.0)
Hypoglycemia	2 (22.2)	0 (0.0)	0 (0.0)	0 (0.0)	0 (0.0)	0 (0.0)
Diabetic ketoacidosis	0 (0.0)	0 (0.0)	0 (0.0)	0 (0.0)	0 (0.0)	0 (0.0)
Pancreatitis	0 (0.0)	0 (0.0)	0 (0.0)	0 (0.0)	0 (0.0)	0 (0.0)
Events leading to LLA	0 (0.0)	1 (16.7)	0 (0.0)	—	—	—
Urinary tract infection	0 (0.0)	2 (33.3)	0 (0.0)	0 (0.0)	1 (5.9)	0 (0.0)
Genital infection	0 (0.0)	0 (0.0)	0 (0.0)	0 (0.0)	1 (5.9)	2 (40.0)
Vitamin D deficiency	3 (33.3)	2 (33.3)	0 (0.0)	0 (0.0)	1 (5.9)	0 (0.0)
Bone fracture	0 (0.0)	0 (0.0)	0 (0.0)	0 (0.0)	0 (0.0)	0 (0.0)

Abbreviations: AE, adverse event; AESI, adverse event of special interest; LLA, lower limb amputation.

^a^Pooled participants from the DINAMO and DINAMO MONO trials.

## Discussion

The American Diabetes Association guidelines recommend screening for T2D in adults aged 35 years and over [11], and studies focusing on young adults with T2D aged 18-35 years are sparse, with only 15% to 20% of all adults with T2D worldwide diagnosed <40 years of age [12]. This study was therefore an opportunity to assess SGLT2 and DPP-4 inhibitor monotherapies in young adults with T2D and to compare these outcomes with adolescents with T2D.

In this analysis of adolescents (aged 10-17) with T2D from the pooled DINAMO and DINAMO MONO trial sample, who were drug-naïve or not on active therapy, neither empagliflozin nor linagliptin treatment showed a difference in treatment failure occurrence vs placebo. However, empagliflozin did reduce treatment failure occurrence vs placebo in young adults aged 18-35 with no antidiabetic background therapy. Treatment failure was also the primary endpoint of the TODAY study, with a similar rate of failure (51.7%) in participants on metformin monotherapy to those on empagliflozin monotherapy (50.0%) [4]. In addition, there was a numerical trend for a reduction in HbA1c in the treatment arms of both the adolescent and young adult study samples vs placebo, with empagliflozin treatment.

There are several limitations of this analysis to consider. Firstly, we acknowledge the low number of participants. Secondly, 1 participant experienced severe AEs (due to coexisting conditions/medications), which disproportionately affected the safety results. Thirdly, due to sitagliptin being the sole DPP-4 inhibitor available at the time of EMPA-REG MONO, different DPP-4 inhibitor comparators were used in young adults and adolescents. Participants of DINAMO MONO and EMPA-REG MONO were also recruited under different conditions. For these reasons direct comparisons between adolescents and young adults should be undertaken with caution. Finally, there was variation in baseline HbA1c values between treatment arms and between adolescents and young adults. While there was a trend for reduction in HbA1c with empagliflozin and linagliptin, or sitagliptin, in both adolescents and young adults, direct comparison of the magnitude of reduction in HbA1c cannot be made since this is correlated to baseline HbA1c [13].

The TODAY study showed that some adolescents (aged 10-17 years) with T2D can be effectively treated with metformin monotherapy in the first year; however, more than half of those on metformin monotherapy failed to maintain glycemic control during the second year of treatment [4, 14]. Further, the TODAY study identified the need for combination antidiabetic therapies, as in that study only the metformin plus rosiglitazone group experienced superior glycemic outcomes. In our study, empagliflozin modestly reduced HbA1c vs placebo in adolescents, with greater reductions seen in young adults. Linagliptin also modestly reduced HbA1c vs placebo in adolescents. Sitagliptin did not meaningfully improve glycemic control vs placebo in young adults, although results were comparable with empagliflozin up to Week 18.

In the DINAMO trial, hypoglycemia was the most frequently reported AE for both the empagliflozin and the linagliptin groups [6]. In this pooled DINAMO and DINAMO MONO sample, there were no events of hypoglycemia or diabetic ketoacidosis in the empagliflozin or linagliptin groups, confirming the established safety profile of these drugs. Both amputations in the participant treated with empagliflozin were suspected to be related to development of peripheral ischemia thromboses, as the participant had multiple thrombotic risk factors (heterozygosity of the factor V Leiden mutation, obesity, and use of systemic hormonal contraception).

## Conclusion

These results highlight the challenges of diabetes monotherapy for children and adolescents with T2D and are consistent with the TODAY study findings. Taken together, these observations reinforce the need to rapidly move from monotherapy for a large proportion of youth with T2D in the early years following diagnosis and that combination therapy should be considered.

## Data Availability

To ensure independent interpretation of clinical study results and enable authors to fulfil their role and obligations under the ICMJE criteria, Boehringer Ingelheim grants all external authors access to relevant clinical study data. In adherence with the Boehringer Ingelheim Policy on Transparency and Publication of Clinical Study Data, scientific and medical researchers can request access to clinical study data, typically, 1 year after the approval has been granted by major Regulatory Authorities or after termination of the development program. Researchers should use the https://vivli.org/ link to request access to study data and visit https://www.mystudywindow.com/msw/datasharing for further information.

## Clinical Trial Information

NCT03429543 (registered June 6, 2018).

## Abbreviations

AE: adverse event
DPP-4: dipeptidyl peptidase-4
FPG: fasting plasma glucose
HbA1c: glycated hemoglobin
SGLT2: sodium-glucose co-transporter-2
T2D: type 2 diabetes
